# Reversal of Atherosclerotic Plaque Growth and Vulnerability: Effects of Lipid-Modifying and Anti-Inflammatory Therapeutic Agents

**DOI:** 10.3390/biomedicines12112435

**Published:** 2024-10-23

**Authors:** Michail I. Papafaklis, Rafail Koros, Grigorios Tsigkas, Antonios Karanasos, Athanasios Moulias, Periklis Davlouros

**Affiliations:** 1 Faculty of Medicine, University of Patras, 26504 Rio, Greece; 2 Cardiology Division, University Hospital of Patras, 26504 Rio, Greece

**Keywords:** atherosclerosis, plaque regression, plaque stabilization, lipid-lowering therapies, coronary artery disease, intravascular ultrasound, optical coherence tomography, intravascular imaging, statins, PCSK9

## Abstract

Atherosclerotic plaque development constitutes the primary substrate of coronary artery disease (CAD) and is the outcome of an intricate process involving endothelial damage, inflammation, and lipid retention. The clinical efficacy of many lipid-lowering therapies in patients with CAD has been well established. Over the past few decades, a substantial and significant advance regarding the use of invasive and non-invasive imaging modalities has been observed. Numerous studies have been conducted using these imaging techniques and have investigated the changes in morphology (e.g., atheroma volume) and composition (e.g., lipid burden, fibrous cap thickness, macrophage accumulation) at the plaque level that explain the improved clinical outcomes by various pharmacological interventions. Lipid-lowering agents, such as statins and proprotein convertase subtilisin/kexin type 9 (PCSK9) inhibitors, demonstrate direct effects on plaque volume and composition that enhance plaque stabilization and/or regression beyond the reduction of low-density lipoproteins. An increasing amount of clinical research is also focused on the role of inflammation in plaque vulnerability and future adverse cardiac events. Consequently, there is a pressing need to explore therapeutic strategies that are capable of disrupting the inflammatory response as well as reducing atheroma burden and modifying high-risk plaque characteristics. This review provides a comprehensive analysis of the current evidence regarding the effects of traditional and novel therapeutic strategies targeting modification of the lipid profile and inflammatory processes on reversing plaque growth and attenuating vulnerable features, thereby promoting plaque stabilization and passivation.

## 1. Introduction

Coronary artery disease (CAD) is the most common cause of death in the United States, as it accounts for approximately 610,000 deaths annually (estimated 1 in 4 deaths) and the third most common cause of death globally [1]. Atherosclerotic plaque development and progression is the pathophysiologic substrate of CAD. Early animal experiments have shown that cessation of fat-rich diets was associated with a decrease in arterial lesions induced by high cholesterol nutrition [2]. The initial stage of the disease presents with intimal thickening due to aggregation of low-density lipoprotein (LDL) molecules in the intima of the arterial wall. The inflammatory process is central in the evolution of atherosclerosis, with lymphocytes (mainly T cells) and monocytes infiltrating the intimal layer, and the formation of foam cells through lipid uptake by macrophages ensues. Macrophage infiltration within lipid pools and subsequent apoptosis of these macrophages leads to the formation of a lipid-rich and acellular necrotic core surrounded by fibrous tissue [3,4].

Seminal work on the histology of atherosclerosis during different stages of the disease has provided the classification of the plaque types [5,6]. Early stages are represented by intimal xanthomas and pathological intimal thickening corresponding to focal accumulations of macrophage foam cells, lipid-laden smooth muscle cells, and extracellular lipid accumulation. Continued disease progression leads to the formation of fibroatheromas, which also include a lipid core, while plaque ageing may lead to fibrocalcified plaques consisting of superficial or deep calcifications. The natural history of the disease also includes complicated plaques defined by disruption of the endothelial surface inducing hematoma or thrombotic debris, thereby leading to acute events. The most common cause of acute coronary syndromes (ACS) is the presence of an intracoronary thrombus superimposed on a complicated atherosclerotic plaque. The three major pathophysiological mechanisms for ACS involve plaque rupture, plaque erosion, and, less frequently, the presence of a calcified nodule. Plaque rupture, the most frequent cause of intracoronary thrombosis, occurs when the fibrous cap of the fibroatheroma is disrupted and separates from the lipid-necrotic core; the thrombogenic lipid core is then exposed to the blood flow [7].

Halting the process of atherosclerosis and the natural history of the disease has been the goal of research efforts for almost a century. It has been widely recognized from animal experiments as early as the 1920s that regression of arterial lesions induced by high cholesterol diets can be accomplished after cessation of cholesterol feeding. This observation has been confirmed in many species, i.e., rabbits, dogs, pigs, and nonhuman primates. Furthermore, studies conducted over the past four decades have already established the close association between atherosclerosis regression with low LDL cholesterol and higher high-density lipoprotein (HDL) cholesterol concentrations [2]. Subsequent experimental studies on monkeys in the 1990s demonstrated a potential benefit from the use of angiotensin-converting enzyme inhibitors and angiotensin II antagonists in inhibiting atheromatous lesions in the aorta in *Cynomolgus* monkeys fed a high-cholesterol diet [8]. Since then, the data on pharmacotherapy with many different agents providing benefit in plaque regression and stabilization have multiplied.

This review aims to give an update on the therapeutic strategies (lipid-lowering and anti-inflammatory) that could promote the stabilization of the atheromatic plaque structure, thereby mitigating plaque rupture/thrombogenicity and minimizing the possibility of acute coronary events.

## 2. Plaque Vulnerability: Unstable Atherosclerotic Lesions

Vulnerable plaques (i.e., prone to rupture) present similar morphologic and functional characteristics as ruptured plaques, with the exception of an intact fibrous cap [9]. In the 1980s, the term vulnerable plaque was initially described as the highly susceptible plaque to rupture, and more recently, Stone et al. correlated vulnerability with the increased possibility of major adverse cardiovascular events [10,11].

A common pathologic feature of vulnerable plaques, firstly identified in autopsy studies, is the presence of reduced cap thickness (typically less than 65 μm) [12]. Cap infiltration by macrophages results in its thinning and potentially rupture due to the secretion of proteolytic enzymes such as matrix metalloproteinases [13]. Other vulnerable plaque characteristics include large size and a large necrotic core covered by the thin cap. Plaques susceptible to rupture have a smaller burden of calcification [14].

Vulnerable plaques, in contrast to stable plaques, are associated with positive remodeling, an outward expansion of the plaque, and a 5% increase in cross-sectional area, preventing the narrowing of the lumen. Due to the frequent presence of compensatory artery remodeling, 90% of TCFAs are found on large plaques with at least intermediate stenosis (>50% area stenosis) [15]. Positive remodeling induces neovascularization by microvessels originated by adventitial vasa vasorum. This results in an adverse effect on plaque stability due to the presence of intraplaque hemorrhage. Intraplaque neovascularization, which correlates with circulating cholesterol levels and neointimal macrophage content, is promoted by hypoxia in the plaque microenvironment and subsequent inflammatory cell death. Intraplaque hemorrhage is provoked by extravasation of erythrocytes from the immature neo-vessels. The free cholesterol contained in erythrocyte membranes contributes to the cholesterol pool in the necrotic core [16,17,18,19].

Thin-cap fibroatheroma has been identified as an adverse predictor of cardiovascular events in patients with non-obstructive lesions in angiography [20]. The COMBINE-OCT FFR (Combined Optical Coherence Tomography Morphologic and Fractional Flow Reserve Hemodynamic Assessment of Non-Culprit Lesions to Better Predict Adverse Event Outcomes in Diabetes Mellitus Patients) study revealed that vulnerable plaques depicted by optical coherence tomography in diabetic patients accounted for over 80% of future adverse cardiovascular events, even though they represent about 25% of angiographically intermediate lesions that are not functionally significant (i.e., negative fractional flow reserve assessment) [21].

Consequently, the hypothesis was created that major acute coronary events arise from lesions that are not necessarily severe, but also mild/moderate coronary stenoses can lead to increased cardiovascular morbidity. The common denominator of these lesions is that they share specific histopathological characteristics. The PROSPECT (Providing Regional Observations to Study Predictors of Events in the Coronary Tree) study was a natural-history study that confirmed the above hypothesis. Data from gray-scale and radiofrequency intravascular ultrasonographic imaging revealed that the presence of a large plaque burden, a small luminal area, or both even in angiographically mild lesions could predict the increased risk of major adverse cardiovascular events within 3 years. Moreover, major events associated with mild lesions occurred at sites identified by the presence of thin-cap fibroatheromas detected by intravascular ultrasonography with virtual histology. These findings support the idea that plaques with morphological features consistent with vulnerable plaques represent the highest-risk clinical phenotype [22].

Another study by Xing et al. managed to clarify the recurrence of major adverse coronary events despite advances in percutaneous coronary intervention (PCI) and in pharmacological strategies. The prevalence of lipid accumulation was considered an indicator of plaque vulnerability and was assessed by measurement of lipid length and lipid arc. These calculations were acquired by OCT imaging. Lipid extent was demonstrated to be a strong predictor of major adverse cardiovascular events during 24-month, 36-month, and 48-month follow-up, confirming the strong clinical association between high-risk vulnerable plaque features of non-culprit lesions and the manifestation of adverse clinical events [23].

## 3. Detection of Vulnerable Plaques: Imaging Modalities

In the past decades, a wide variety of coronary imaging modalities have emerged and enabled the comprehensive analysis of atherosclerotic plaques. Invasive techniques primarily include intravascular ultrasound (IVUS), optical coherence tomography (OCT), and near-infrared spectroscopy (NIRS), while recent technological evolution has enabled non-invasive evaluation of plaque features using computed tomography coronary angiography (CTCA) and positron emission tomography (PET).

IVUS has been considered the gold standard invasive diagnostic tool used for the quantification of coronary plaques [24]. IVUS allows the precise visualization of all three layers of the coronary artery wall, and thus, accurate assessment of several parameters used to estimate atherosclerosis burden can be performed by IVUS. Calculation of atheroma area in each cross-sectional image and of total atheroma volume (TAV) can be performed based on the pullback speed during image acquisition. Percent atheroma volume (PAV) can be recorded as the percent of the volume of the elastic external membrane (EEM) occupied by atheroma. IVUS also facilitates distinguishing fibrous and fatty components of plaque, the necrotic core, and calcium and helps to classify plaques qualitatively according to the tissue echogenicity of the lesion (compared to that of adventitia) to soft, fibrous, calcified, and mixed plaque [25].

OCT is an invasive imaging method that provides higher resolution than IVUS (axial resolution of 10–20 μm versus 80–250 μm, respectively) but at the cost of lower depth penetration (0.1 to 2.0 mm). Consequently, a more detailed view of the abluminal coronary vessel structure is generated. Although OCT presents a more limited quantification of lipid content and plaque volume due to reduced depth penetrance, a highly reliable detection of thin-cap fibroatheroma can be provided based on the presence of a thin fibrous cap (<65–75 μm) overlying a signal-poor lesion with diffuse border (lipid arc >90°) [26]. Augmented macrophage accumulation, considered to be indicative of an inflamed unstable plaque, is visualized by OCT as a linear high-intensity signal with high attenuation on the lesion surface [27]. Microchannels indicating the presence of neovessels can also be depicted by OCT as non-signal tubuloluminal structures [28].

NIRS is an invasive imaging modality that uses a light source in the near infrared spectrum and analyses the amount of light reflected to determine the chemical composition of the tissue, enabling semiquantitative measurement of lipid content (cholesterol and cholesteryl esters) [29]. Culprit segments are more likely to exhibit a larger lipid core burden index (LCBI; above 400 measured by NIRS), which has been considered to be a reliable element of plaque instability [30].

The latest technological developments in CTCA providing higher resolution have enabled the non-invasive visualization of all major epicardial arteries with assessment of stenosis severity and both qualitative and quantitative information on plaque morphology; plaque type can be distinguished as calcified, non-calcified, or mixed. Positive remodeling, low plaque attenuation, and “spotty” calcification are CTCA findings that correlate closely with increased cardiovascular risk. Nevertheless, it should be noted that CTCA presents a higher number of artefacts and continues to have lower resolution compared to intravascular modalities [31].

Positron emission tomography (PET) is a non-invasive technique based on the use of radiotracers (e.g., 18F-FDG, a radiolabeled glucose analogue). The uptake of such tracers by the arterial wall can be used as a surrogate for metabolic activity and aids in the detection of regions presenting plaque inflammation and calcification [32,33].

## 4. Statin Treatment

**Figure 1:**
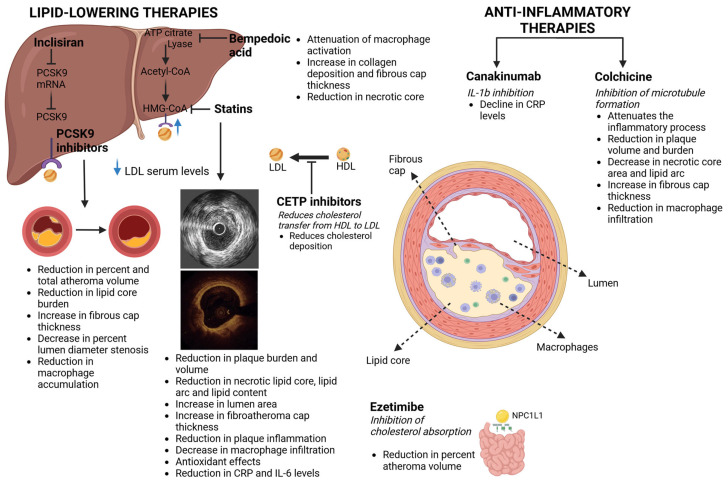
Lipid-lowering (focus on cholesterol) and anti-inflammatory therapeutic agents with their principal mechanism of action, and summary of their effects on atherosclerotic plaques leading to plaque regression and stabilization.

Statins have been widely recognized as the cornerstone of lipid-lowering treatment. Their function is based on inhibition of 3-hydroxy-3-methylglutaryl–coenzyme A reductase (HMG-CoA reductase inhibitors), the enzyme that mediates the cholesterol synthesis pathway. The consequent increased LDL receptor expression at the surface of the hepatocytes leads to increased uptake of LDL from the blood and decreased plasma concentrations of LDL- and other ApoB-containing lipoproteins, including triglyceride-rich particles [34] (Figure 1).

Several randomized trials and meta-analyses have examined the contribution of statins in reducing adverse cardiovascular events. The decrease in LDL-C levels achieved by statins has been established as a determinant factor for mitigating cardiovascular risk, and it is consistent with the evidence derived from genetic and clinical studies regarding the role of LDL in the pathogenesis of atherosclerosis [35]. HPS (Heart Protection Study), 4S (Scandinavian Simvastatin Survival Study), and WOSCOPS (West of Scotland Coronary Prevention Study) were three of the first large-scale landmark trials published in the 1990s confirming the clinical value of statin treatment over a median 5-year follow-up. In the HPS trial, the addition of 40 mg simvastatin daily in patients with diabetes, CAD, or other occlusive artery disease was associated with a reduction in myocardial infraction, revascularization, or stroke by 25%. Results from the 4S study were in total accordance with HPS, reflecting an explicit benefit with respect to morbidity and mortality in patients with pre-existing CAD. Additionally, the WOSCOPS (West of Scotland Coronary Prevention Study) trial verified the advantageous effects of pravastatin on reducing hard endpoints defined by cardiovascular death and myocardial infarction in high-risk patients in the context of primary prevention [36,37,38].

Cholesterol Treatment Trialists’ (CTT) Collaboration, established in 1997, performed a large meta-analysis of 14 randomized trials, including 90,056 patients. The data revealed a lower 5-year incidence of major cardiovascular events, need for revascularization, and stroke by approximately one-fifth per 1 mmol/L (38.67 mg/dL) reduction in LDL cholesterol. The results had already been beneficial since the first year, as a 23% and 21% decrease was present for major coronary events and major vascular events, respectively, over the first-year follow-up; the benefit was evident regardless of the initial patient’s LDL concentration [39]. CTT collaboration also demonstrated that a further reduction in LDL cholesterol adopting more intensive statin therapeutic regimens provides an incremental efficacy profile in terms of reduced occlusive vascular events [40]. Later, results of the randomized PROVE-IT (Pravastatin or Atorvastatin Evaluation and Infection Therapy) and TNT (Treating to New Targets) trials led to the establishment of reduced target goals from 100 to 70 mg/dL in secondary prevention patients [41,42].

### 4.1. Statin Trials on Plaque Regression

Several studies have traditionally confirmed the great effect of statins on the reduction in cardiovascular morbidity and mortality. Among the pleiotropic effects of statins, stabilization of atherosclerotic plaques, making them less prone to rupture, seems to be a convincing explanation for their clinical benefits. The effect of statin treatment on plaque stability and regression has been widely studied in numerous heterogenous studies. Scientific evidence attests that statins induce morphological modifications in plaque composition. Assessment with a variety of imaging modalities, including OCT, IVUS, NIRS, CTCA, and FDG-PET, demonstrated a reduction in total atheroma volume and necrotic lipid core and an increase in fibrous thickness [43,44,45,46] (Table 1).

Tagaki et al. conducted in 1997 the first randomized trial that displayed the results of statin treatment on plaque progression. Pravastatin was compared to placebo, and IVUS was utilized to evaluate the atheroma volume. The pravastatin group was observed to considerably lessen the progression of atherosclerotic lesions compared to placebo [47].

Several studies investigated the interaction between statin treatment and disease progression. A small-scale study by Corti et al. underlined the long-term effects of simvastatin on the percentage dimension change in atherosclerotic lesions located on the aorta and carotid arteries, as detected by high-resolution magnetic resonance imaging. The earliest change identified was a decrease in plaque dimensions, i.e., vessel wall thickness and vessel wall area. Thus, the most striking observation was the beneficial arterial remodeling which was also accompanied by a slight but significant increase in lumen area, detected after a 24-month follow-up. Reduction in total and LDL plasma cholesterol levels by 23% and 38% (*p* < 0.01), respectively, were evident after 6 weeks of treatment. These findings supported the maintenance of statin treatment for longer periods [48].

Compared to pravastatin, atorvastatin is more lipophilic with higher bioavailability, has extensive metabolism via CYP3A4, minimal renal excretion, and a longer half-life (≈14 h vs. 1.5–2 h of pravastatin), making it more potent and less affected by food for absorption [49]. The novelty of the REVERSAL (Reversal of Atherosclerosis with Aggressive Lipid Lowering) trial, published back in 2004, was that the intensity of statin therapy serves a pivotal role in plaque regression, and high-intensity statins can inhibit more effectively atherosclerotic progression compared to moderate-intensity statins. The study demonstrated that patients with CAD who were administered intensive statin treatment with 80 mg atorvastatin daily compared to moderate treatment with 40 mg pravastatin exhibited a slower rate of atheroma volume progression detected by IVUS combined with a greater mean LDL cholesterol reduction. Of note, a 36.4% C-reactive protein (CRP) reduction observed in the intensive treatment group was found to be an independent predictor of atherosclerosis regression [50].

**Table 1:** Major studies investigating the effect of statins on plaque regression and stabilization.

Study	Year	Comparison Groups	Imaging Modality	Patients (n)	Follow-Up Duration	Endpoint(s)
REVERSAL [50]	2005	Pravastatin 40 mg vs. atorvastatin 80 mg	IVUS	654	18 months	Change in atheroma volume: +2.7% (*p* = 0.001) in pravastatin arm vs. −0.4% (*p* = 0.98) in the atorvastatin arm, (*p* = 0.02) Nominal change in atheroma volume: +4.4% (*p* = 0.001) in pravastatin arm vs. −0.9% (*p* = 0.72) in the atorvastatin arm, (*p* = 0.02) Change in PAV: +1.6% (*p* < 0.001) in pravastatin arm vs. +0.2% (*p* = 0.18) in the atorvastatin arm, (*p* < 0.001)
ASTEROID [51]	2006	Rosuvastatin 40 mg: baseline vs. follow-up	IVUS	507	24 months	Mean (SD) change in PAV for the entire vessel: −0.98% (*p* < 0.001 vs. baseline) Mean (SD) change in atheroma volume in the most diseased 10 mm subsegment: −6.1 mm^3^ (*p* < 0.001 vs. baseline) Total atheroma volume: 6.8% median reduction (*p* < 0.001 vs. baseline)
JAPAN-ACS [52]	2009	Pitavastatin vs. atorvastatin	IVUS	307	8 to 12 months	Mean percentage change in PV was −16.9% in pitavastatin vs. −18.1% in atorvastatin group (*p* = 0.5)
ATHEROMA [53]	2009	10 mg vs. 80 mg atorvastatin	USPIO-enhanced MRI	47	12 weeks	Change in USPIO-defined inflammation: 80 mg group: ΔSI 0.13 (*p* = 0.0003) at 6 weeks and ΔSI 0.2 (*p* < 0.0001) at 12 weeks 10 mg group: ΔSI 0.004 (*p* = 0.15) at 6 weeks and ΔSI –0.03 (*p* = 0.3) at 12 weeks
SATURN [54]	2011	Atorvastatin 80 mg vs. rosuvastatin 40 mg	IVUS	1039	104 weeks	PAV: –0.99% with atorvastatin and –1.22% with rosuvastatin (*p* = 0.17) Normalized TAV: −6.39 mm^3^ with atorvastatin and −4.42 mm^3^ with rosuvastatin (*p* = 0.01). PAV change: 63.2% with atorvastatin and 68.5% with rosuvastatin (*p* = 0.07) TAV change: 64.7% with atorvastatin and 71.3% for rosuvastatin (*p* = 0.02)
YELLOW [55]	2013	Rosuvastatin 40 mg vs. standard therapy	Νear-infrared spectroscopy	87	7 weeks	LCBI4mm max median change: –149.1 in rosuvastatin group vs. 2.4 in standard therapy (*p* = 0.01)
EASY-FIT [56]	2014	Atorvastatin 20 mg vs. atorvastatin 5 mg	OCT	70	12 months	Fibrous cap thickness change: +69% with 20 mg/day atorvastatin vs. +17% with 5 mg/day atorvastatin (*p* < 0.001)
STABLE [57]	2016	Rosuvastatin 40 mg vs. rosuvastatin 10 mg	IVUS	312	12 months	Percent NC volume change within the target segment: –3.72 ± 0.71 in the 40 mg vs. –2.17 ± 0.7% (*p* = 0.22) in the 10 mg group Percent fibrofatty volume change: 3.80 ± 0.71 in the 40 mg vs. 1.69 ± 0.74% (*p* = 0.009) in the rosuvastatin 10 mg Percent fibrous volume: –0.07 ± 0.58 in the 40 mg vs. 0.34 ± 0.60% in the 10 mg rosuvastatin (*p* = 0.79) Percent dense calcium volume change: –0.02 ± 0.35 in the 40 mg vs. 0.82 ± 0.37 in the 10 mg rosuvastatin group (*p* = 0.19)
PARADIGM [58]	2018	Statin-taking vs. statin-naive patients	CTCA	1255	Median 3.4 years between 2 CTCAs	PAV progression: 1.76 ± 2.40% per year in statin-taking patients vs. 2.04 ± 2.37% (*p* = 0.002) in statin-naive patients Calcified PAV progression: 1.27 ± 1.54 vs. 0.98 ± 1.27% per year in statin-naive patients (*p* < 0.001) Non-calcified PAV progression: 0.49 ± 2.39 vs. 1.06 ± 2.42% per year (*p* < 0.001) Annual new HRP features: 0.9 in statin-taking patients vs. 1.6% per year in statin-naive patients (*p* < 0.001)
ESCORT [59]	2018	Early vs. late post-ACS pitavastatin	OCT	53	36 weeks	FCT change: from 140 mm to 160 mm in the early statin group (*p* = 0.017) vs. from 135 to 130 mm in the late statin group (*p* = 0.020)
IBIS-4 [60]	2019	Rosuvastatin 40 mg: baseline after ACS vs. follow-up	OCT and IVUS	103	13 months	Minimum FCT increase: from 64.9 ± 19.9 to 87.9 ± 38.1 μm (*p* = 0.008) Macrophage line decrease: from 9.6 ± 12.8 to 6.4 ± 9.6° (*p* < 0.0001) Mean lipid arc decrease: from 55.9 ± 37 to 43.5 ± 33.5° (*p* = 0.013)

ACS: acute coronary syndrome, CTCA: computerized tomography coronary angiography, FCT: fibrous cap thickness, HRP: high-risk plaque, IVUS: intravascular ultrasound, LCBI4mm max: lipid-core burden index at the 4-mm maximal segment, MRI: magnetic resonance imaging, NC: necrotic core, OCT: optical coherence tomography, PAV: percent atheroma volume, PV: plaque volume, SD: standard deviation, TAV: total atheroma volume, USPIO: ultrasmall superparamagnetic iron oxide, ΔSI: change from baseline in signal intensity.

After the interesting results of the REVERSAL trial, the significant impact of aggressive lipid-lowering therapy on inhibiting atherosclerosis progression in statin-naïve patients was highlighted by the outcomes of the ASTEROID (A Study to Evaluate the Effect of Rosuvastatin on Intravascular Ultrasound-Derived Coronary Atheroma Burden) trial in 2006. ASTEROID was the largest trial, including 507 patients, that investigated the effect of high dose intensive lipid-lowering treatment on modifying the atheroma volume during a two-year follow-up. Atheroma was assessed with IVUS at baseline and after two years. Patients were assigned to rosuvastatin or placebo groups. Administration of 40 mg rosuvastatin induced a reduction in plaque atheroma volume for the entire vessel at a mean (SD) of −0.98% (3.15%), with a median of −0.79% (97.5% CI, −1.21% to −0.53%; *p* < 0.001 vs. baseline). Furthermore, the mean (SD) change in atheroma volume in the most diseased 10 mm subsegment was −6.1 (10.1) mm^3^, with a median of −5.6 mm^3^ (97.5% CI, −6.8 to −4.0 mm^3^; *p* < 0.001 vs. baseline). Rosuvastatin 40 mg treatment showed efficacy on LDL-C reduction (by 53%) and HDL-C increase (by 15%) similar to that of 80 mg atorvastatin in the PROVE-IT (Pravastatin or Atorvastatin Evaluation and Infection Therapy) and TNT (Treating to New Targets) studies. The fact that 75% of enrolled patients achieved a mean LDL-C level of less than 70 mg/dL (LDL-C reduction levels to a mean of 60.8 mg/dL) during follow-up explains that the attainment of stricter LDL-C target goals leads to effective atheroma burden reduction. It is worth noting that the reported LDL-C levels decrease along with the magnitude of HDL-C increase were the greatest ever observed in a statin atherosclerosis progression trial [51].

Similar evidence on the inhibition of coronary atherosclerosis progression was revealed by the SATURN (Study of Coronary Atheroma by Intravascular Ultrasound: Effect of Rosuvastatin versus Atorvastatin) trial. The study design was based on the comparison of two potent statins, administered at the highest dose over a 24-month follow-up (rosuvastatin 40 mg was compared with atorvastatin 80 mg daily). Both regimens proved a considerable and nearly equivalent effect on coronary artery regression. Although the primary efficacy point, percent atheroma volume, was not significantly different between the groups, rosuvastatin administration achieved a TAV reduction to a greater extent (−6.39 vs. −4.42 mm^3^, *p* = 0.01). The atorvastatin group presented a marginally lower decrease in LDL-C levels (62.5 mg/dL vs. 70.2 mg/dL, *p* < 0.001) and increase in HDL-C levels (48.6 mg/dL vs. 50.4 mg/dL, *p* = 0.01) than the rosuvastatin group. These data indicate that CAD can regress if the favorable levels of LDL and HDL cholesterol that were attained with statin therapy in this study are achieved [54].

The YELLOW (Reduction in Yellow Plaque by Aggressive Lipid-Lowering Therapy) trial, published in 2013, sought to demonstrate regression of atherosclerosis in terms of markedly reduced lipid content, which is a feature of plaque vulnerability. The study was conducted among patients with multivessel CAD who were randomized to intensive statin therapy or standard lipid-lowering treatment and were serially assessed at baseline and 7 weeks using NIRS. The study met its primary endpoint and demonstrated that short-term intensive statin therapy significantly reduced lipid content compared to the standard group [55].

In the STABLE (Statin and Atheroma Vulnerability Evaluation) trial, high-intensity lipid-lowering therapy by rosuvastatin facilitated plaque regression by 67% in accordance with other trials such as the ASTEROID and SATURN. Percent necrotic core volume, total atheroma volume, and the frequency of thin-cap fibroatheroma defined by VH-IVUS were effectually decreased at one year. However, in contrast to other trials, plaque regression was evident in a similar range regardless of rosuvastatin dose (10 mg vs. 40 mg rosuvastatin). Lower body mass index, a higher level of hs-CRP at baseline, and a larger percent necrotic core volume at baseline were associated with greater necrotic core reduction. Plaque regression was proportionally correlated with hs-CRP reduction, implying an anti-inflammatory mechanism of rosuvastatin treatment, whereas no direct association with LDL reduction was established [57].

Similar regressive effects were observed in the JAPAN-ACS (Japan Assessment of Pitavastatin and Atorvastatin in Acute Coronary Syndrome) trial for patients who were evaluated with IVUS after an ACS. Treatment with 4 mg pitavastatin or 20 mg atorvastatin on a daily basis both resulted in significant plaque volume depletion at 8–12 months without inferiority shown for any of the two regimens. In accordance with the STABLE (Statin and Atheroma Vulnerability Evaluation) trial, plaque regression was observed irrespective of the broad spectrum of LDL-C reduction [52].

A meta-analysis including 31 studies (4997 patients) supported the fact that the lower the LDL goal achieved by the patients, the better the outcome in terms of preventing further progression of the disease and reduction in the atheroma volume. The study evaluated the impact of statin therapy on coronary atheroma volume and correlated it with the percentage change in LDL and HDL levels. In all selected studies, quantitative assessment of atherosclerotic plaque was performed with IVUS. Statin treatment resulted in significantly lower TAV and PAV at follow-up. Plaque regression was linked directly with a proportional change in LDL and HDL cholesterol levels. TAV reduction was achieved when LDL at follow-up was decreased at a value less than 80 mg/dL and HDL was greater than or equal to 45 mg/dL, whereas PAV reduction was established at an LDL value less than 90 mg/dL. The analysis suggested that plaque regression in patients with coronary disease required an LDL level below 80 mg/dL and HDL above 45 mg/dL to occur [61].

### 4.2. Effect of Statins on High-Risk Plaque Characteristics

The effects of lipid-lowering treatment and specifically statins on atherosclerosis regression go beyond the beneficial relationship between the decrease in LDL cholesterol and the reduction in lipid content and atheroma volume. Pleiotropic effects of statins have been reflected on their impact on several high-risk plaque features.

In a study conducted 20 years ago, pravastatin treatment for 3 months was associated with reduced lipid content, oxidized LDL immunoreactivity, cell death, and inflammatory cell and matrix metalloproteinase (MMP)-2 immunoreactivity, along with an increase in tissue inhibitor of metalloproteinases (TIMP)-1 immunoreactivity and interstitial collagen content in human carotid plaques, indicating the anti-inflammatory and antioxidant effects of statins on human carotid plaque composition [62]. In the clinical setting, the effect of high dose pitavastatin on increasing fibrous cap thickness was shown in the ESCORT (Effect of Early Pitavastatin Therapy on Coronary Fibrous Cap Thickness Assessed by Optical Coherence Tomography in Patients with Acute Coronary Syndrome) trial [59], which supported the initiation of statin treatment after an ACS. The conclusion that can be drawn is that early initiation of statin treatment after an ACS is associated with substantial benefit regarding plaque stabilization and reduction in vulnerable plaques in non-culprit vessel areas. Furthermore, these outcomes might reflect the inflammatory environment after an ACS characterized by an increase in circulating concentrations of inflammatory cytokines and biomarkers and the vulnerability of plaques across the entire coronary tree.

The impact of aggressive statin therapy on inflammation was studied in more detail later in the ATHEROMA (Atorvastatin Therapy: Effects on Reduction of Macrophage Activity) study, demonstrating compelling results. Supporting the notion that macrophage infiltration is a principal predisposing factor for plaque rupture, the study used ultrasmall superparamagnetic iron oxide (USPIO)-enhanced magnetic resonance imaging to detect carotid plaque inflammatory lesions in humans. It was found that patients on 80 mg atorvastatin had a greater reduction in USPIO-defined plaque inflammation at 6 and 12 weeks compared to patients on 10 mg atorvastatin, establishing an association of statin therapy with reducing plaque inflammation and vulnerability [53].

The reduction in the inflammatory process of atherosclerotic plaques has also been demonstrated in the EASY-FIT (Effect of Atorvastatin Therapy on the Fibrous Cap Thickness in Coronary Atherosclerotic Plaque as Assessed by OCT) study, which included 70 patients who presented with unstable angina pectoris. Patients were randomized to a higher (20 mg) or lower (5 mg) dose of atorvastatin, and the composition of atheromatous plaque was assessed over a 12-month follow-up period. A more prominent increase in fibrous cap thickness and lipid core attenuation was demonstrated with the use of time-domain OCT. Furthermore, the stabilization of atheromatous plaques combined with the anti-inflammatory advantages that higher dose atorvastatin treatment showed was demonstrated by not only the reduced hs-CRP and IL-6 values but also by a significant reduction in malondialdehyde (MDA)-LDL (a cardiovascular risk factor independent of overall LDL) and MMP-9 [56].

Beyond an increase in fibrous cap thickness indicating plaque stabilization, reduced macrophage accumulation reflecting attenuated inflammation was also the conclusion by the first serial OCT evaluation of plaque composition in the non-infarct-related coronary arteries of patients with ST-segment elevation myocardial infarction (STEMI) under treatment with rosuvastatin [60].

In the much larger patient population of the PARADIGM (Progression of AtheRosclerotic PlAque Determined by Computed TomoGraphic Angiography IMaging) study, including patients without a history of CAD, statin treatment demonstrated a salutary effect on coronary arterial remodeling using non-invasive imaging with coronary CT. Remarkable outcomes were observed describing the statin-induced transformation of plaque morphology with increased plaque calcification and a reduction in high-risk plaque features such as low-attenuation plaque and positive arterial remodeling [58].

## 5. Ezetimibe

Ezetimibe monotherapy leads to reduced serum LDL-C levels by approximately 20% without any safety or tolerability concerns. Ezetimibe exerts its action through inhibition of cholesterol absorption at the brush border of the small intestine via the sterol transporter named Niemann-Pick C1-like intracellular cholesterol transporter 1 (NPC1L1) [63] (Figure 1). After oral administration, it is rapidly absorbed and broadly metabolized (>80%) to the pharmacologically active ezetimibe-glucuronide. Following enterohepatic recycling and slow elimination, total ezetimibe concentrations reach a maximum 1–2 h post-administration; the estimated terminal half-life of ezetimibe and ezetimibe–glucuronide is approximately 22 h [64].

In the randomized PRECISE-IVUS (Plaque Regression With Cholesterol Absorption Inhibitor or Synthesis Inhibitor Evaluated by Intravascular Ultrasound) trial [65], combination therapy of ezetimibe with statin revealed regression effects in contrast to statin monotherapy. Volumetric analysis by IVUS in patients undergoing PCI showed superiority for plaque regression (absolute change in percent atheroma volume [PAV]: −1.4% vs. −0.3%; *p* = 0.001) along the coronary vessels; coronary plaque regression was also more frequent in the combination therapy group (78 vs. 58%, *p* = 0.004). Moreover, the demonstrated LDL-C reduction (63.2 mg/dL vs. 73.3 mg/dL; *p* < 0.001) confirmed the association between LDL-C reduction and atherosclerosis regression as evidenced by other studies. It should be highlighted that the favorable effects of dual lipid-lowering treatment were more prominent in the high-risk ACS patients. Furthermore, the above data confirm and provide an explanation for the beneficial clinical outcomes presented by the IMPROVE-IT (Improved Reduction of Outcomes: Vytorin Efficacy International Trial) trial. A small trial including STEMI patients, the OCTIVUS (Ezetimibe In Addition To Atorvastatin Therapy On The Plaque Composition in Patients with Acute Myocardial Infarction) study, affirmed the induced plaque regression after the one-year combination therapy with atorvastatin 80 mg and ezetimibe 10 mg in parallel with a 25% LDL-C level decrease. Nevertheless, plaque characterization analysis by IVUS did not show a significant modification in the necrotic core [66]. The lack of any difference in plaque composition despite plaque regression with the combination therapy (ezetimibe/statin) was also observed in the similarly designed HEAVEN (Virtual Histology Evaluation of Atherosclerosis Regression During Atorvastatin and Ezetimibe Administration) study [67].

## 6. PCSK9 Inhibitors

The proprotein convertase subtilisin/kexin type 9 (PCSK9) is secreted by hepatocytes and promotes the degradation of the LDL receptors, thereby decreasing the levels of the receptors at the cell surface. The PCSK9 inhibitors are fully humanized monoclonal antibodies that bind and inhibit free plasma PCSK9 protein. The PCSK9 inhibition permits a greater number of LDL receptors to remain intact on the cell surface, and circulating LDL plasma levels decline [68]. Additionally, PCSK9 inhibition downregulates inflammatory processes. This is justified by the mediating role of PCSK9 in the activation of nuclear factor kappa beta (Nf-κB) and enhanced expression of pro-inflammatory genes [69], while the degradation of apolipoprotein E receptor 2 is promoted [70]. Finally, PCSK9 inhibition presents favorable effects in suppression of endothelial dysfunction and apoptosis [69]. Several reports support the benefits derived from PCSK9 inhibition, including a decrease in cardiovascular risk accompanied by plaque regression and stabilization (Figure 1; Table 2).

Early results based on IVUS virtual histology were published by the ATHEROREMO-IVUS (European Collaborative Project on Inflammation and Vascular Wall Remodeling in Atherosclerosis-Intravascular Ultrasound) study that first demonstrated a direct correlation of PCSK9 serum levels and plaque stabilization. Coronary angiography and IVUS analysis were performed in 581 patients with stable CAD or recent acute coronary events. There was a linear relationship between lower PCSK9 serum levels and a decreased percent of necrotic core plaque tissue, suggesting that PCSK9 inhibition could emerge as a therapeutic approach targeted at limiting plaque vulnerability; these findings were independent of plasma LDL levels or statin administration [71].

In line with the preliminary outcomes of the ATHEROREMO-IVUS study, the randomized GLAGOV (Global Assessment of Plaque Regression with PCSK9 Antibody as Measured by Intravascular Ultrasound) trial supported the effect of PCSK9 inhibitors on atherosclerosis regression in patients who do not achieve a reduction in cardiovascular events or an adequate LDL-C decrease in spite of optimal statin treatment. Overall, 968 patients were randomized to subcutaneous monthly administration of 420 mg evolocumab or placebo on top of statin agents; IVUS was used for plaque volume assessment. All patients had angiographically confirmed CAD, had received a stable statin dose for over a month, and presented LDL-C plasma levels over 80 mg/dL or 60–80 mg/dL in the presence of either 1 major (non-coronary atherosclerotic cardiovascular disease, acute coronary syndrome, or type 2 diabetes mellitus) or 3 minor (current smoking, hypertension, low HDL-C, family history of premature CAD, age [men ≥ 50 y; women ≥ 55 y], CRP ≥ 2 mg/L) risk factors. After 76 weeks, the primary and secondary endpoints were met in favor of evolocumab demonstrating greater atherosclerosis regression. Percentage atheroma volume was significantly reduced by 0.95% in the evolocumab group, while the change was negligible in the placebo group. Total atheroma volume was substantially reduced in the evolocumab group compared with the neutral effect in the placebo group (2.9 vs. 0.4%; *p* < 0.05). Additionally, lower plasma LDL-C levels were presented in the treatment arm (36.6 vs. 93.0 mg/dL; difference –56.5 mg/dL, 95% CI –59.7 to –53.4, *p* < 0.001). However, PCSK9 inhibition had no effect on plaque composition [72].

The comparison of adding evolocumab with solely high-intensity statin treatment on atherosclerosis regression was also explored in the HUYGENS (High-Resolution Assessment of Coronary Plaques in a Global Evolocumab Randomized Study) trial [73]. The trial design was divergent from that of the GLAVOV trial since it included 161 patients after a recent ACS and plaque composition was evaluated by OCT. After 52 weeks of treatment with evolocumab 420 mg, a greater absolute increase in minimum fibrous cap thickness (primary efficacy point) resulted compared to placebo (42.5 vs. 21.5 mm, *p* = 0.015); percent change in minimum fibrous cap thickness was 81.8% in the treatment group and 44.3% in the placebo group (*p* = 0.04). Favorable changes by evolocumab were also evidenced by a wider increase in average minimum fibrous cap thickness of all images and a decrease in maximum lipid arc (−57.5° vs. −31.4°; *p* = 0.04).

**Table 2:** Major studies investigating the effect of PCSK9 inhibitors on plaque regression and stabilization.

Study	Year	Comparison Groups	Imaging Modality	Patients (n)	Follow-Up Duration	Endpoint(s)
GLAGOV [72]	2016	Evolocumab vs. placebo	IVUS	968	78 weeks	PAV change: −0.95% with evolocumab and +0.05% with placebo (*p* < 0.001) Normalized TAV change: −0.9 mm^3^ with placebo and −5.8 mm^3^ with evolocumab (*p* < 0.001)
ODYSSEY J-IVUS [74]	2019	Alirocumab vs. standard therapy	IVUS	206	36 weeks	Percent change in normalized TAV: −3.1 ± 1.0% with standard therapy vs. −4.8 ± 1.0% with alirocumab (*p* = 0.23)
HUYGENS [73]	2022	Evolocumab vs. placebo	OCT	161	58 weeks	Minimum FCT: +42.7 mm in the evolocumab vs. +21.5 mm in the placebo group (*p* = 0.015) Maximum lipid arc: −57.5 in the evolocumab vs. −31.4° in the placebo group (*p* = 0.04) Macrophage index: −3.17 in the evolocumab vs. −1.45 mm in the placebo group (*p* = 0.04) Percent atheroma volume change: −2.29 ± 0.47% in the evolocumab group vs. −0.61 ± 0.46% in the placebo group (*p* = 0.009)
PACMAN-AMI [75]	2022	Alirocumab vs. placebo	IVUS-NIRS-OCT	300	52 weeks	Mean change in percent atheroma volume: −2.13% with alirocumab vs. −0.92% with placebo (*p* < 0.001) Mean change in maximum LCBI within 4 mm: −79.42 with alirocumab vs. −37.60 with placebo (*p* = 0.006) Mean change in minimal FCT: +62.67 μm with alirocumab vs. +33.19 μm with placebo (*p* = 0.001)
YELLOW III [76]	2023	Evolocumab: baseline vs. follow-up	IVUS-NIRS-OCT	300	26 weeks	FCT change: from 70.9 to 97.7 µm (*p* < 0.001) Max LCBI-4mm: from 306.8 to 213.1 (*p* < 0.001)
PACMAN-AMI lesion-level analysis [77]	2024	Alirocumab or placebo	IVUS-NIRS-OCT	245	52 weeks	Lesion-level mean change in PAV was −4.86% with alirocumab vs. −2.78% with placebo (*p* < 0.001) At the MLA site: mean change in PAV was −10.14% with alirocumab vs. −6.70% with placebo (*p* < 0.001) MLA increased by 0.15 mm^2^ with alirocumab and decreased by 0.07 mm^2^ with placebo (*p* = 0.04) Lipid-rich plaque phenotype at follow-up: 61.8% in the alirocumab arm vs. 41.8% in the placebo arm (*p* = 0.03) Fibrous/fibrocalcific plaque phenotype at follow-up: 30.8% in the alirocumab arm vs. 8.1% in the placebo arm (*p* = 0.02)

ACS: acute coronary syndrome, CTCA: computerized tomography coronary angiography, FCT: fibrous cap thickness, HRP: high-risk plaque, IVUS: intravascular ultrasound, LCBI: lipid-core burden index, MLA: minimum lumen area, MRI: magnetic resonance imaging, NC: necrotic core, NIRS: near-infrared spectroscopy, OCT: optical coherence tomography, PAV: percent atheroma volume, PV: plaque volume, SD: standard deviation, TAV: total atheroma volume.

In accordance with evolocumab studies, plaque regression was more dominant in patients with acute MI treated with alirocumab compared to placebo with respect to the primary endpoint of percent change in atheroma volume (–2.13% with alirocumab versus –0.92% with placebo) detected by serial multimodality intravascular imaging in non-culprit coronary arteries. In detail, the recent PACMAN-AMI (Effects of the PCSK9 Antibody Alirocumab on Coronary Atherosclerosis in Patients With Acute Myocardial Infarction) trial randomized 300 participants on high-intensity statin treatment who had undergone PCI due to acute MI to subcutaneous alirocumab 150 mg every 2 weeks or placebo; comprehensive analysis of plaque phenotype and composition was generated by three imaging modalities (IVUS-NIRS and OCT) performed at baseline and 52 weeks [75]. The larger absolute PAV reduction in the alirocumab group in comparison to other trials was explained by the fact that approximately 90% of the assigned patients were statin naive. Alirocumab administration was also associated with a remarkably greater reduction in the maximum lipid-core burden index evaluated by NIRS and a greater increase in minimal fibrous cap thickness evaluated by OCT. A latest post hoc analysis focusing on lesions with more severe pathology demonstrated larger effects on atheroma volume reduction (entire lesion: change in PAV −4.9% with alirocumab vs. −2.8% with placebo, *p* < 0.001; at the minimum lumen area: −10.1 vs. −6.7%, *p* < 0.001, respectively) compared to the initial vessel-level analysis; thus, advanced lesions with higher plaque burden are more predisposed to benefit from aggressive lipid-lowering intervention [77]. Both PCSK9-inhibitor trials, HUYGENS and PACMAN-AMI, investigating patients with an ACS, demonstrated consistency regarding the plaque stabilizing effects of PCSK9 inhibition therapy on top of statins after short-term follow-up.

Beyond the impact on morphological characteristics, the hemodynamics of a coronary stenosis and any benefit in reducing inducible ischemia are also important in the clinical setting. The PACMAN-AMI QFR sub-analysis investigated the impact of alirocumab in addition to high-intensity lipid-lowering therapy on coronary hemodynamics estimated by quantitative flow ratio (QFR) and on lumen anatomy by three-dimensional quantitative coronary angiography %diameter stenosis in non-obstructive non-culprit lesions [78]. Alirocumab treatment was not associated with a statistically significant effect on the primary endpoint (QFR increase) compared with placebo, even though a slight benefit was observed over 12 months as QFR numerically increased in 53% of patients with alirocumab versus 40% of patients with placebo, while the magnitude of the decrease in % diameter stenosis was minimal. Thus, it is speculated that the principal effect of PCSK9 inhibitors on halting atherosclerosis progression is caused by plaque stabilization rather than lumen increase followed by improvement in coronary hemodynamics.

Similar results with the previous studies, but in a different reference population that included stable CAD patients, were recently presented from the YELLOW III (Reduction in Yellow Plaque by Aggressive Lipid-Lowering Therapy III) study investigators [76]. Aggressive lipid-lowering therapy for 26 weeks with a PCSK9-inhibitor regimen, on top of a statin, led to a significant improvement in minimal fibrous cap thickness by OCT (+26.8 ± 22.3 μm, *p* < 0.001), reduction in lipid core burden index at the maximal 4 mm segment assessed by NIRS (−93.7 ± 140.5, *p* < 0.001), and reduction in atheroma volume measured by IVUS in angiographically non-obstructive lesions. Peripheral blood mononuclear cell gene expression analysis is also part of the YELLOW III study with the goal of creating predictive models for detecting patients who demonstrate the greatest response to PCSK9 inhibition therapy regarding changes in plaque morphology.

The Japanese ODYSSEY-J-IVUS (Evaluation of Effect of Alirocumab on Coronary Atheroma Volume in Japanese Patients Hospitalised for Acute Coronary Syndrome with Hypercholesterolaemia) randomized trial published contradictory results not supporting the magnitude of the plaque stabilizing effects of PCSK9 inhibitors as demonstrated by the above trials. Alirocumab 75 mg treatment in addition to low-intensity statin therapy was compared to statin-only therapy in 206 patients hospitalized (1–12 months ago) with ACS and residual hyperlipidemia (plasma LDL-C level > 100 mg/dL). The primary endpoint, defined by the percentage change in normalized total atheroma volume measured by IVUS, did not reach the desired statistical significance, although a slight decrease in atheroma volume was observed. The non-significant results of this trial could be interpreted by the shorter treatment period (9 months) and the smaller sample size, which could affect the power of the study to detect a significant impact on atherosclerosis regression [74].

## 7. Other Lipid-Modifying Therapies: Targeting Triglycerides

Hypertriglyceridemia has been considered a source of potentially increased cardiovascular risk. Although pharmacological agents lowering LDL-C have shown efficacy on atherosclerosis progression, drugs lowering triglycerides have not been established as a fundamental treatment for prevention of cardiovascular events [79].

The GISSI-Prevenzione (Gruppo Italiano per lo Studio della Sopravvivenza nell’Infarto miocardico) trial showed that dietary supplementation with n-3 polyunsaturated fatty acids (PUFA) remarkably reduced cardiovascular events in patients after myocardial infarction [80]. The benefits of icosapent ethyl, a purified eicosapentaenoic acid ethyl ester, were supported by the REDUCE-IT (Reduction of Cardiovascular Events with Icosapent Ethyl-Intervention Trial) trial, which showed a 25% reduction in the incidence of cardiovascular morbidity in 8000 enrolled patients with elevated triglyceride levels at high cardiovascular risk [81].

Although there are several data indicating the clinical benefits provided by n-3 PUFA, there is not a great deal of scientific evidence supporting their impact on atherosclerosis progression. When fatty acid therapy was tested in addition to high-intensity statin, an increase in fibrous cap thickness was observed by OCT in patients with an ACS who had plaques with rather low cap thickness and thus higher risk [82]. The CHERRY (Combination therapy of eicosapentaenoic acid and pitavastatin for coronary plaque regression evaluated by integrated backscatter intravascular ultrasonography) and EVAPORATE (Effect of Vascepa [icosapent ethyl] on Improving Coronary Atherosclerosis in People With High Triglycerides Taking Statin Therapy) trials also provide ambitious results indicating that these agents could reduce inflammation and reverse endothelial dysfunction. In the CHERRY trial [83], a randomized comparison between combination therapy of eicosapentaenoic acid 1800 mg plus pitavastatin 4 mg daily or pitavastatin 4 mg daily alone was performed; 193 patients with a history of CAD undergoing PCI were included. At a 6- to 8-month follow-up, plaque volume and composition were evaluated by integrated backscatter-IVUS, and a significant reduction in normalized TAV was demonstrated equivalently in both arms. However, TAV decrease was more prominent in the group of patients who received additionally eicosapentaenoic acid. No significant difference was observed in terms of dense fibrosis and calcification volumes. Lipid volume reduction was detected only in the group of patients who received the combination therapy. Another interesting finding of the trial was that the reversal of the lipid volume was more frequent in patients with ACS rather than in patients with stable CAD.

The EVAPORATE trial published in 2020 confirmed the outcomes of the CHERRY trial, but using a different imaging modality [84]. The investigators aimed to examine whether icosapent ethyl promoted plaque regression in comparison to placebo. Plaque volume was analyzed by CTCA in 80 patients with documented coronary atherosclerotic lesions, who had already received statin treatment and presented high triglyceride levels. Over a follow-up of 18 months, the icosapent ethyl group was associated with remarkably reduced low attenuation plaque, fibrofatty volume, and fibrous volume. It should be noted that there was concern regarding the received mineral oil by the placebo group due to potential adverse effects on apolipoprotein-B and LDL-C and the subsequent impact on the final outcomes. Nevertheless, there was not any difference in plaque progression between mineral oil and cellulose-based placebos, which could explain a potential underestimation of plaque regression in the placebo group.

Pemafibrate is a selective peroxisome proliferator-activated receptor α modulator (PPARα) modulator that reduces triglyceride levels and improves lipid levels; it has the advantage of a better safety profile for liver and renal function [85]. However, only animal study data are available regarding the impact of pemafibrate on atherosclerotic plaques. In a study with LDL receptor knock-out (LDLR-KO) pigs and a balloon injury model, inhibition of the inflammatory process in coronary vessel atherosclerosis was revealed [86]. In the clinical setting, in the randomized PROMINENT (The Pemafibrate to Reduce Cardiovascular Outcomes by Reducing Triglycerides in Patients with Diabetes) trial, the administration of pemafibrate did not achieve the primary efficacy endpoint of reducing cardiovascular events during approximately 3 years [87].

Niacin (nicotinic acid) induces intracellular degradation of apoB lipoproteins and reduces both degradation of HDL-C and hepatic triglyceride synthesis [88]. In a small study, ALPINE SVG (Atherosclerosis Lesion Progression Intervention Using Niacin Extended Release in Saphenous Vein Grafts), there were neutral data regarding the impact of niacin on percent atheroma volume in saphenous vein grafts imaged by OCT [89].

## 8. Lipids Modification by HDL Increase: CETP Inhibitors

A potential protective role of higher HDL levels against cardiovascular mortality has been proposed by observational data [90]. Cholesteryl ester transfer protein (CETP) inhibitors increase HDL-C plasma levels and prevent cholesterol transfer from high-density lipoproteins to low-density lipoproteins, thereby decreasing LDL-C levels (Figure 1). However, this advantageous effect has not been portrayed in clinical trials. The oldest early-terminated ILLUMINATE (Investigation of Lipid Level Management to Understand its Impact in Atherosclerotic Events) trial failed to show a beneficial impact of torcetrapib on reducing cardiovascular mortality and morbidity [91]. There was also no clinical benefit in patients with ACS in the dal-OUTCOMES (Effects of Dalcetrapib in Patients with a Recent Acute Coronary Syndrome) trial comparing dalcetrapib with placebo [92]. No improvement in cardiovascular risk was revealed in high-risk patients in the ACCELERATE (Evacetrapib and Cardiovascular Outcomes in High-Risk Vascular Disease) trial for patients treated with evacetrapib [93]. However, in the randomized HPS3/TIMI55 REVEAL (Effects of Anacetrapib in Patients with Atherosclerotic Vascular Disease) trial, the addition of anacetrapib at a dose of 100 mg daily to intensive statin therapy for a longer follow-up of 4 years achieved a lower rate of major coronary events than placebo-controlled patients with established cardiovascular disease [94].

Very few data have been exhibited about the CETP effect on plaque composition. In the ILLUSTRATE (Lipid Level Management Using Coronary Ultrasound to Assess Reduction of Atherosclerosis by CETP Inhibition and HDL Elevation) trial [95], 910 patients were assigned to torcetrapib plus atorvastatin versus atorvastatin alone, and plaque volumetric analysis by IVUS was performed. After 24 months, the addition of torcetrapib to standard treatment failed to show a significant outcome for the primary efficacy measure of change in percent atheroma volume. In view of these disappointing results, the development of torcetrapib was terminated in 2006.

The phase 2b dal-PLAQUE (Safety and efficacy of dalcetrapib on atherosclerotic disease using novel non-invasive multimodality imaging) trial presented more promising results regarding the safety and efficacy profile of CETP inhibitors on plaque regression [96]. With reference to the MRI-based carotid indices, dalcetrapib resulted in a smaller increase in total vessel area over 6 months compared to placebo (*p* < 0.05). Furthermore, the analysis of ^18^F-FDG-PET data in the carotids suggested that an anti-inflammatory mechanism mediates the favorable role of dalcetrapib in vascular remodeling. All these results were accompanied by an elevation in HDL-C plasma levels by 31%.

The randomized CHI-SQUARE (Can HDL Infusions Significantly QUicken Atherosclerosis REgression) and CARAT (Effect of Serial Infusions of CER-001, a Pre-beta High-Density Lipoprotein Mimetic, on Coronary Atherosclerosis in Patients Following Acute Coronary Syndromes in the CER-001 Atherosclerosis Regression Acute Coronary Syndrome Trial) trials aimed to investigate the contribution of an intravenous HDL-C mimic agent (CER 100) infusion to plaque regression in statin-treated patients following an ACS. Both trials revealed neutral effects on atherosclerosis reduction [97,98]. Similar to previous trials, in the MILANO-PILOT (Effect of Infusion of High-Density Lipoprotein Mimetic Containing Recombinant Apolipoprotein A-I Milano on Coronary Disease in Patients With an Acute Coronary Syndrome) trial, no advantage was presented by the administration of HDL mimetic containing apolipoprotein A-1 Milano (MDCO-216) in patients with ACS on top of statins [99]. Lastly, in the ERASE (Effect of HDL on Atherosclerosis-Safety and Efficacy) trial, the post-ACS patients who received reconstituted HDL did not demonstrate an incremental reduction in plaque volume measured by IVUS compared to placebo-controlled patients [100].

## 9. Anti-Inflammatory Agents

### 9.1. Colchicine

Inflammation plays a key role in atherosclerosis progression. Consequently, questions arise regarding the potential impact on the mechanism of atherosclerosis from drugs aimed at suppressing inflammation. Colchicine is an old and inexpensive medication that has been proposed as a potential plaque stabilization agent, mainly due to its multiple anti-inflammatory properties. Colchicine’s primary action is polymerization of tubulin and inhibition of microtubule formation, resulting in a suppressed inflammatory process (Figure 1). In experimental models, colchicine was associated with inhibition of foam cell transformation of macrophages after in vitro atherogenic stimuli with oxidized LDL. Attenuated inflammatory response induced decreased necrotic core area and reduced lipid accumulation of carotid plaque in mice [101].

The results of the LoDoCo (Low-Dose Colchicine) trial in 2013 reflected a favorable clinical profile related to colchicine administration in patients with stable CAD over a 3-year follow-up. Non-stent-related acute coronary events were reduced in the colchicine group [102]. Similar results were observed for major adverse cardiovascular events in the LoDoCo 2 trial. The effect of colchicine on mitigation of the inflammatory process was considered the principal cause of these outcomes [103].

The data regarding the effect of colchicine on plaque regression are limited. In a nonrandomized observational study, which used CTCA [104], 80 patients with recent ACS (within a month) were treated with 0.5 mg per day colchicine plus optimal medical treatment or solely optimal medical treatment. CTCA was performed after one year and suggested a substantially beneficial correlation of low dose colchicine with improved plaque composition; low attenuation plaque volume was diminished (15.9 vs. 6.6 mm^3^; *p* < 0.01). However, no significant difference was noted regarding the total atheroma volume between the two groups. The reversal of plaque burden induced by colchicine was in line with a decline in CRP levels, highlighting the critical role of diminishing inflammation for plaque regression.

The latest COLOCT (Effect of Colchicine on Coronary Plaque Stability in Acute Coronary Syndrome as Assessed by Optical Coherence Tomography) trial highlighted the impact of colchicine on plaque stability through inhibition of vascular inflammation [105]. This is the first study demonstrating that the addition of colchicine to standard medical therapy remarkably improved plaque morphology assessed by OCT. The study included 128 patients with ACS with lipid-rich plaque (lipid pool arc > 90°) detected by OCT and randomized them in a 1:1 ratio to receive either colchicine (0.5 mg once daily) or placebo for one year. The primary end point, the change in the minimal fibrous cap thickness from baseline, was significantly increased in the colchicine group (51.9 versus 87.2 μm; *p* < 0.01). Furthermore, reductions in average lipid arc and mean angular extension of macrophages were observed, as well as reduced high-sensitivity C-reactive protein and myeloperoxidase levels. These findings show a strong correlation of the anti-inflammatory properties of colchicine with beneficial effects on plaque passivation in a very high-risk population.

These promising data indicate the necessity for further future studies. The ongoing COCOMO-ACS (Colchicine for coronary plaque modification in acute coronary syndrome) study, which will recruit patients with recent ACS to receive colchicine or placebo, is going to provide further evidence with respect to the efficacy of colchicine therapy on non-culprit atheromatous lesions assessed by OCT [106].

### 9.2. Canakinumab: Targeting Cytokines

Cytokines (e.g., IL-1 and IL6) are central to the inflammatory process. The CANTOS (The Canakinumab Anti-inflammatory Thrombosis Outcome Study) study included high-risk patients presenting a history of myocardial infraction and investigated whether canakinumab, a human monoclonal antibody functioning as an interleukin-1 inhibitor, could lead to a benefit in clinical outcomes. A total of 741 patients were enrolled and randomized to a 1:1:1 ratio to receive placebo, canakinumab at a dose of 150 mg, or canakinumab at a dose of 300 mg. Canakinumab administration was ensued by a decline in high-sensitivity CRP and interleukin-6 whereas no change occurred in terms of LDL and HDL levels. The primary endpoint defined by first occurrence of nonfatal myocardial infarction, any nonfatal stroke, or cardiovascular death was reduced by 15% in the 150 mg canakinumab group (3.86 vs. 4.50 events per 100 person-years in the placebo group; *p* = 0.021), confirming that treating inflammation could lead to a reduction in cardiovascular events. No statistically significant reduction was shown with respect to the primary endpoint between 150 mg and 300 mg canakinumab (3.90 events per 100 person-years). Although no important decrease in lipid levels occurred in this trial, the extent of benefit on cardiovascular events with canakinumab was similar to that presented by monoclonal antibodies targeting PCSK9. These data suggest that therapeutic agents targeting inflammatory pathways are expected to provide benefit for slowing atherosclerosis progression [107].

## 10. Emerging Lipid-Modifying Therapies

New therapies are being introduced with an optimistic outlook for modifying the lipid profile. Bempedoic acid has been shown to decrease LDL-C levels by inhibiting ATP citrate lyase in the liver, reducing cholesterol biosynthesis, and upregulating LDL receptors (Figure 1). Bempedoic acid is administered as a pro-drug and is converted to its active metabolite mostly in the liver and not in skeletal muscles, and has the benefit of practically no muscle side effects. Clinical trials have tested its use both as monotherapy and as complementary therapy in patients who do not reach their target goals (LDL > 70 mg/dL) on a maximally tolerated statin dose with or without additional lipid-lowering agents [108,109]. Overall, bempedoic acid is associated with an approximately 15–20% decrease in LDL-C levels compared to placebo and, when used as monotherapy, a significant reduction in cardiovascular events. Furthermore, the anti-inflammatory effects of the drug in the clinical setting have been supported by a significant reduction in high-sensitive CRP levels [108]. On the atherosclerotic plaque level, attenuation of inflammation due to reduced macrophage activation and a significant reduction in lipid accumulation with smaller necrotic cores along with increased collagen deposition and fibrous cap thickness have been observed in an experimental study with mice [110]. However, documentation regarding the effects of bempedoic acid on human plaque characteristics is still lacking.

Inclisiran is an interfering RNA (siRNA) molecule that targets PCSK9, and it is anticipated to be strongly associated with plaque regression (Figure 1). In a pooled analysis of the ORION-9, ORION-10, and ORION-11 (inclisiran for subjects with atherosclerotic cardiovascular disease [ASCVD] or ASCVD-risk equivalents and elevated LDL-C) studies, inclisiran administration twice a year in patients with ASCVD and elevated LDL levels led to approximately 50% decrease in LDL-C levels at 510 days compared to placebo (*p* < 0.0001). No side effects concerning kidney, liver, and muscle toxicity were observed [111]. Except for the clinical efficacy of inclisiran, recent preliminary data have demonstrated its contribution to reducing atherosclerotic plaque lipid content and LDL-C levels when added in a triple therapy regimen in patients for whom statin and/or ezetimibe therapy alone was insufficient. Serial evaluation by NIRS measurements resulted in a significant reduction in the maximum lipid-core burden index within 4 mm (maxLCBI4 mm) in the triple therapy group at 15 months [112].

Based on the hypothesis that HDL constitutes a beneficial factor for halting the progression of atherosclerosis, reconstituted HDL infusions have been considered as an emerging therapeutic strategy. In an early study, short-term infusions of a moderate dose of CSL112, a human plasma-derived apolipoprotein A-I, were associated with notable improvements in plaque characterization indices by IVUS and a lumen-based coronary score assessed by quantitative coronary angiography, although no difference in percentage change in atheroma volume or nominal change in plaque volume was observed in IVUS measurements compared to placebo. Furthermore, an adverse safety profile regarding liver function was noted with a higher dose [100]. Evidence from experimental studies focused on histopathological findings after CSL112 infusion suggests that the restoration of cholesterol efflux through CSL112 promotes the stabilization of vulnerable atherosclerotic plaques via multiple anti-inflammatory and immune-regulatory mechanisms [113]. The recent AEGIS-II (ApoA-I Event Reducing in Ischemic Syndromes II) trial (phase 3, multicenter, randomized, placebo-controlled) focused on the clinical efficacy and safety of CSL112 administration in patients after ACS with respect to the frequency of recurrent cardiovascular events. No statistically significant difference in the risk of experiencing a primary end-point event at 90 days and 180 days of follow-up was observed between the CSL112 and placebo groups [114]. There was no imaging study for assessing plaque regression. Elevating HDL remains a promising therapeutic strategy in vascular disease, and further investigation, including trials focusing on clinical outcomes, is warranted.

## 11. Conclusions

A diverse array of therapeutic interventions is available to mitigate the risk of cardiovascular events by reversing plaque growth and inducing plaque stabilization. Robust outcome data have already established a direct association between plaque regression/stabilization and a reduction in CV events in some types of therapies. The application of imaging modalities that can monitor atherosclerotic plaque volume and assess composition/characteristics offers a more precise approach for guiding therapeutic decisions. Current data support the simultaneous use of therapeutic agents that target different pathways and can potentially maximize the clinical impact on reducing adverse cardiac events.
